# Understanding Human Factors Challenges on the Front Lines of Mass COVID-19 Vaccination Clinics: Human Systems Modeling Study

**DOI:** 10.2196/39670

**Published:** 2022-11-10

**Authors:** Ryan Tennant, Moses Tetui, Kelly Grindrod, Catherine M Burns

**Affiliations:** 1 Department of Systems Design Engineering Faculty of Engineering University of Waterloo Waterloo, ON Canada; 2 School of Pharmacy University of Waterloo Kitchener, ON Canada; 3 Department of Epidemiology and Global Health Umea University Umea Sweden

**Keywords:** cognitive work analysis, contextual design, COVID-19, decision making, health care system, pandemic, vaccination clinics, workplace stress

## Abstract

**Background:**

Implementing mass vaccination clinics for COVID-19 immunization has been a successful public health activity worldwide. However, this tightly coupled system has many logistical challenges, leading to increased workplace stress, as evidenced throughout the pandemic. The complexities of mass vaccination clinics that combine multidisciplinary teams working within nonclinical environments are yet to be understood through a human systems perspective.

**Objective:**

This study aimed to holistically model mass COVID-19 vaccination clinics in the Region of Waterloo, Ontario, Canada, to understand the challenges centered around frontline workers and to inform clinic design and technological recommendations that can minimize the systemic inefficiencies that contribute to workplace stress.

**Methods:**

An ethnographic approach was guided by contextual inquiry to gather data on work as done in these ad-hoc immunization settings. Observation data were clarified by speaking with clinic staff, and the research team discussed the observation data regularly throughout the data collection period. Data were analyzed by combining aspects of the contextual design framework and cognitive work analysis, and building workplace models that can identify the stress points and interconnections within mass vaccination clinic flow, developed artifacts, culture, physical layouts, and decision-making.

**Results:**

Observations were conducted at 6 mass COVID-19 vaccination clinics over 4 weeks in 2021. The workflow model depicted challenges with maintaining situational awareness about client intake and vaccine preparation among decision-makers. The artifacts model visualized how separately developed tools for the vaccine lead and clinic lead may support cognitive tasks through data synthesis. However, their effectiveness depends on sharing accurate and timely data. The cultural model indicated that perspectives on how to effectively achieve mass immunization might impact workplace stress with changes to responsibilities. This depends on the aggressive or relaxed approach toward minimizing vaccine waste while adapting to changing policies, regulations, and vaccine scarcity. The physical model suggested that the co-location of workstations may influence decision-making coordination. Finally, the decision ladder described the decision-making steps for managing end-of-day doses, highlighting challenges with data uncertainty and ways to support expertise.

**Conclusions:**

Modeling mass COVID-19 vaccination clinics from a human systems perspective identified 2 high-level opportunities for improving the inefficiencies within this health care delivery system. First, clinics may become more resilient to unexpected changes in client intake or vaccine preparation using strategies and artifacts that standardize data gathering and synthesis, thereby reducing uncertainties for end-of-day dose decision-making. Second, improving data sharing among staff by co-locating their workstations and implementing collaborative artifacts that support a collective understanding of the state of the clinic may reduce system complexity by improving shared situational awareness. Future research should examine how the developed models apply to immunization settings beyond the Region of Waterloo and evaluate the impact of the recommendations on workflow coordination, stress, and decision-making.

## Introduction

### Background

The concept of mass immunization was first introduced to the world in 1805, and it involved successfully vaccinating 100,000 people in Mexico for smallpox, albeit with significant logistical hurdles to bring the vaccine across the Atlantic Ocean [1]. More than 217 years later, when the global population faces its largest pandemic with COVID-19, mass immunization has never been a more critical public health activity [2]. However, during the current pandemic, mass immunization remains a multifaceted global health care challenge [3-5].

One significant logistical challenge for mass immunization is coordinating workflow in an environment where health care professionals can administer vaccines quickly and safely. The setting for such a large-scale public health activity is a unique health care context requiring significant planning and preparation among many individuals [6,7]. For example, mass vaccination clinics often combine the expertise of pharmacists, nurses, physicians, nonclinical staff, and volunteers working closely together [8]. Additionally, mass vaccination clinics are usually established in various settings, including schools, large vacant stores, city halls, shopping centers, places of worship, community centers, friendship centers, convention centers, sports arenas, and colleges or universities [6-10]. While these environments are unlikely to be designed to support vaccinating hundreds to thousands of individuals per day, implementing mass vaccination clinics in these settings is an effective way to immunize communities quickly and safely, especially during a pandemic [11].

Despite the effectiveness of COVID-19 mass vaccination clinics in supporting public health, they can be classified as complex health care delivery systems, partly resulting from vaccine brands that require ultra-low temperature storage and adequate time for thawing and mixing before administration [12-14]. They are also tightly coupled systems [15], where dependent and interconnected components, such as vaccine waste and preparation rates, can easily cause a chain reaction that impacts clinic flow [12-14], especially when a vaccine is scarce. However, the impact of this coupling is lessened when there is a surplus supply. Given the multitude of factors involved with operating a mass vaccination clinic in the era of COVID-19 [13], stress points and logistical challenges on the frontlines quickly become apparent [12,13,16].

Few scholarly articles have evaluated mass vaccination clinic design, implementation, and operation, to improve the workload of frontline staff. Some articles examining this topic have focused on developing or implementing clinic simulations and models to improve operating efficiency [17,18] or optimize the geographic placement of clinics [19]. Although the study of vaccine clinics is an emerging research area, few studies have used collaborative human factors–related methodologies to improve process inefficiencies related to clinic design [20]. While vaccine clinic culture and the interconnections among health care workers play critical roles during the evolving COVID-19 pandemic [12], to the best of the authors’ knowledge, a human systems approach has not been applied to analyze the operation of mass COVID-19 vaccination clinics.

### Objective

The complexities associated with multidisciplinary teams working in nontraditional large-scale immunization environments provide a critical opportunity to apply a systems approach to improve these work domains. A systems approach involving human factors modeling and analyses can identify areas and opportunities for improving team and organizational performance, as evidenced by the approach’s usefulness in evaluating the interconnections and relationships within other aspects of the COVID-19 pandemic [21,22]. Therefore, the primary objective of this study was to systematically assess work as done in mass vaccination clinics, using a human factors approach [23], specifically through ethnography and a modified contextual design framework [24], substituting a tool from cognitive work analysis [25].

While primarily guided by the contextual design framework for its usefulness in driving system design [26,27], this study presents workplace models highlighting the relationships, constraints, and stress points related to mass vaccination clinic flow, developed artifacts, culture, physical layouts, and decision-making activities. Insights from this study can inform ways to minimize systemic inefficiencies for the coordinated preparation and delivery of vaccines by health care teams through technology and system design recommendations. Additionally, these results may inform public health decisions on developing or implementing tools and systems required to support frontline workers in response to the global challenge of coordinating and planning mass immunization events. Finally, this research further supports the importance of applying a human factors approach in complex health care settings and advocates for its application to understand and improve similar activities requiring quick and safe delivery of coordinated public health services to large populations.

## Methods

### Overview

This study used an ethnographic approach to collect data on human factors challenges faced by frontline workers operating mass COVID-19 vaccination clinics [28]. First, data collection and observations were guided by contextual inquiry at vaccination clinics in the Region of Waterloo. The collected data were subsequently organized and consolidated into the workplace categorizations within the contextual design framework: flow, artifacts, cultural, and physical [24]. Finally, the data were transformed into generalized representations using each workplace model from the framework to visualize the human system interconnections. Control task analysis was substituted from the cognitive work analysis framework, replacing the sequence model from contextual design. Instead, a decision ladder model was developed based on the skill-, rule-, and knowledge-based framework by Rasmussen [29].

### Contextual Inquiry and Design

The contextual design framework is rooted in systems design and centered around workers. Hence, this methodology was chosen to provide a holistic understanding of the human factors challenges of mass COVID-19 vaccination clinics [26,30-32]. The initial step involves contextual inquiry, a participatory technique combining observations while engaging workers with their tasks. Therefore, while the researchers (RT and MT) observed each clinic, they also asked the clinic staff questions to gain expert knowledge about their roles and responsibilities, and understand how they may be influenced by other factors in the workplace [24,33].

The contextual design framework was modified in this study by substituting the sequence model with control task analysis, using the decision ladder model by Rasmussen [29]. This decision ladder model was 1 of 9 related models of naturalistic decision-making identified in 1989, including the cognitive continuum theory by Hammond and the recognition-primed decision model by Klein [34]. The presented models similarly proposed that knowledge from prior experiences was the mechanism influencing decision-making performance. While the sequence model from contextual design supports mapping the order of tasks and stages where decisions occur, the decision ladder model by Rasmussen was selected because it enables a rich understanding of the traversal among data processing and knowledge states throughout decision-making activities [25,35]. Moreover, the analysis method specifically identifies how a worker’s cognition may move between nonsequential steps [36], similar to the model by Klein. We used the model by Rasmussen to capture vaccination clinic decision-making, exploiting the structure of the decision ladder to facilitate mapping heuristic pathways [36], which may be used to inform public health on improvements to operational guidance and system recommendations.

A guide was developed to record observation data (Table 1), including 4 sections aligned with the modeling frameworks [28,37]. Observed decision-making activities related to vaccine preparation and client intake were documented within all areas of the guide to inform the decision ladder modeling.

**Table 1:** Mass vaccination clinic observation guide.

Model (objectives)	Guiding questions
Flow (communication and coordination)	What are the different roles that clinic staff play? What are the responsibilities for each role? How do staff use the clinic space to coordinate? How do staff use artifacts to coordinate? Who does staff give information to? In what form? Who does the communication go to and from? Is the communication important or an interruption? What are the different roles that clinic staff play?
Artifacts (physical components that support the work)	What is the structure and organization of work items used? What information is there to use? How is it used? What informal and formal notes do staff make? How is information presented? Do they need to customize something for their workspace? How are the artifacts used to support the work?
Cultural (constraints on the work from policy/culture/values)	What is the tone of the clinic? What are the policies and constraints? How are they recorded? What are the clinic staff’s attitudes, feelings, and beliefs? Do attitudes, feelings, and beliefs change over time?
Physical (physical structure of the work environment)	What is the layout and physical location of the clinic? Where are the tools and artifacts used? What is the organization of the workstations? Do staff workstations follow the clinic workflow? Do clinic staff need to relocate often to work?

### Observation Process and Ethics

During the observation period, the researchers (RT and MT) hung posters about observations taking place for a vaccine clinic research project. They also wore institution-branded clothing and name tags to identify themselves among clients and clinic staff. The researchers took notes following the observation guide, which were stored electronically on a secured server for access by the research team. Photos of artifacts and physical workspaces were taken to complement the written notes.

The research team also worked and volunteered for the Region of Waterloo, developing an emic perspective of operating a mass vaccination clinic during the initial COVID-19 immunization campaign. One researcher (CMB) volunteered within the clinics, supporting client flow with other volunteers, security staff, and clinic staff. Another researcher (KG) was the lead pharmacist on the vaccination task force for the Region of Waterloo and worked as a vaccine lead and immunizer in multiple clinics. The third researcher (MT) was involved with activities related to community outreach for vaccination in underserved populations. The fourth researcher (RT) was a volunteer and a pharmacy assistant, gaining first-hand experience tracking and drawing up the COVID-19 vaccines.

### Ethics Approval

This study was reviewed and received approval from 2 research ethics committees: (1) the University of Waterloo research ethics and safety committee (#43288) and (2) the Tri-Hospital Research Ethics Committee (#2021-0735). The Region of Waterloo Public Health and Emergency Services, Grand River Hospital, and the Centre for Family Medicine Family Health Team also approved the study.

### Data Analysis

The observation notes for each participating mass vaccination clinic in this study supported data source triangulation to develop consolidated contextual design models that holistically described the workplace [38]. Combining research expertise in health care systems, public health, and cognitive engineering methods in human factors, the research team reviewed and provided input to iterate on and refine the models, supporting an interdisciplinary analysis. The researchers met weekly throughout the observation period to reflect on and discuss the observation data, where the initial consolidated models were developed by 1 researcher (RT).

## Results

### Clinic Demographics

Between May and June 2021, the research team observed 6 mass vaccination clinics in the Region of Waterloo (Table 2). Approximately 27 hours were spent observing and interacting with the clinic staff at Clinic #1 and 8.5 hours at Clinic #2. For Clinics #3, #4, #5, and #6, the researchers observed the working environments and interacted with the staff for 10, 5, 5, and 16 hours, respectively.

**Table 2:** Mass vaccination clinic characteristics.

Clinic #	Environment type	Average clients/day^a^	Clinical team
1	University building	≤1000	Family health clinic
2	Vacant commercial warehouse	≤2000	Regional hospital
3	Unfinished public health building	≤1500	Public health
4	High school gymnasium^b^	≤500	Public health
5	Public health building^b^	≤500	Public health
6	Conference center^b^	≤5000	Public health

^a^Estimated value during the observation period.

^b^Primarily weekend-only clinics during the observation period.

### Flow Model

**Figure 1:**
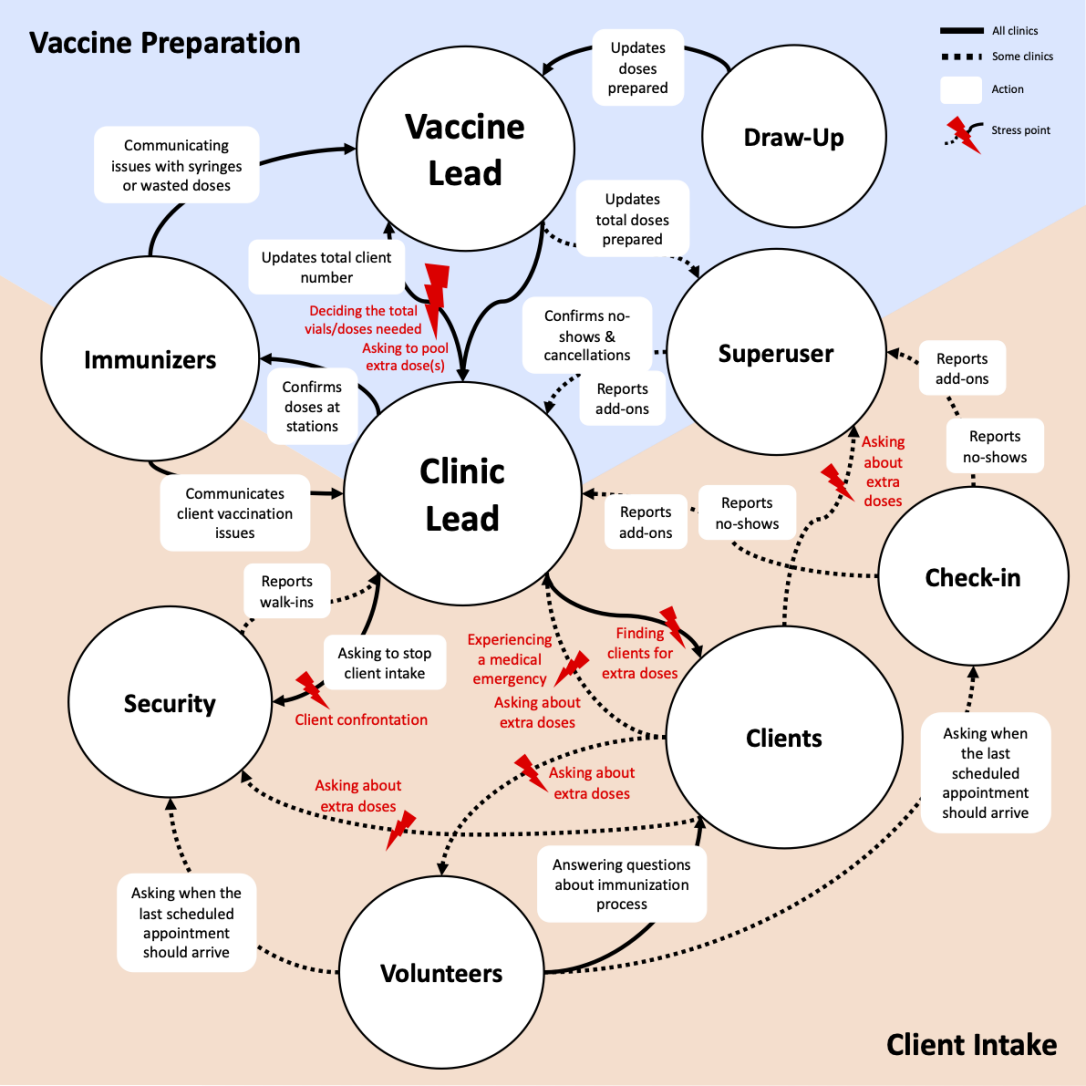
Consolidated flow model for mass vaccination clinics.

Several stakeholders held multiple responsibilities contributing to workflow coordination of the mass vaccination clinics (Multimedia Appendix 1). In Figure 1, the consolidated flow model reveals stakeholder interrelations and influences between tasks and responsibilities while highlighting stress points and challenges in coordinating shared situational awareness among decision-makers. Multimedia Appendix 2 displays a more detailed version of Figure 1.

Information flowed among all stakeholders within the mass vaccination clinics. Salient challenges were observed between the clinic lead, superusers, and the vaccine lead, who continuously made vaccine preparation decisions to serve the constantly changing number of expected clients. However, errors in determining the accurate number of clients or prepared doses could lead to a system with higher gain, where calculation errors or unexpected cancellations result in unanticipated surplus of extra doses, causing increased stress about potential vaccine waste.

Loosening the tightly coupled vaccine preparation and client intake information was often approached through time buffering. For example, when approximately 50 expected clients remained, the clinic lead would halt client flow into the clinic. The team leads would then count the number of prepared doses remaining with immunizers and any doses still being prepared. The objective was to determine how many doses were still needed or if there might be extra doses. However, sudden unexpected changes in the number of clients and extra doses could still destabilize the system after counting, risking a surplus or shortage of doses. Regaining certainty of client intake and vaccine preparation information required a recount of available doses when client intake resumed or when initiating another pause, which could disrupt client flow.

### Artifacts Model

Several artifacts were observed in each mass vaccination clinic to achieve a similar intent, with a visual record of client intake and vaccine preparation changes. The different artifacts aimed to support the cognitive task of knowing how many doses were needed as early as possible but focused on tracking the details of client intake or vaccine preparation. The primary artifacts observed were categorized into 2 models: one for the vaccine lead and the other for the clinic lead.

#### Vaccine Lead Artifacts Model

**Figure 2:**
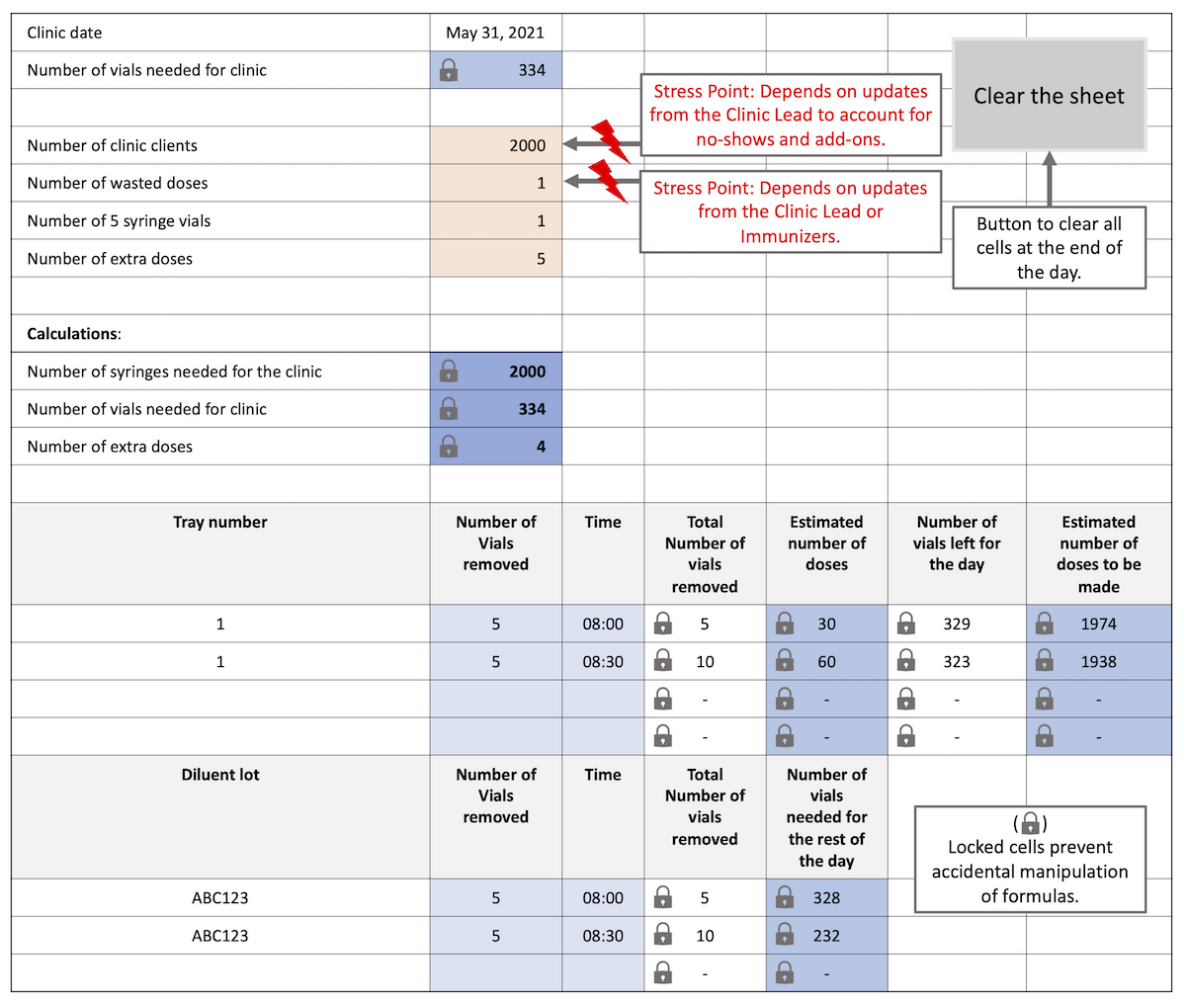
Vaccine lead artifact model for vaccine and client tracking.

Vaccine leads typically modified paper-based or digital artifacts to keep running totals of vaccine preparation that could be reported on public health forms at the end of the clinic day. A consolidated model combining aspects of digital and paper-based artifacts (Figure 2) is depicted as a digital spreadsheet.

While the tracking artifacts evolved by requiring more information as the pandemic changed, both artifacts were modified differently (writing in the margins or adding new cells). The terms used in these artifacts also differed between clinics. For example, extra doses created from the residual volumes of fully prepared vials were either referred to as a “pooled dose” or a “residual dose.” For clinics that provided 2 brands of vaccines, the vaccine tracking artifact would be duplicated to keep the dose tracking separated.

The initial calculations for the expected number of doses required information on the total number of clients. These data were partly obtained from the client booking website. However, the booking website could not account for client add-ons or walk-ins. Challenges resulted from not having timely updated information on client intake data from the clinic lead and the calculations from their separate tracking artifact. Unknown wasted doses at immunization stations further contributed to vaccine preparation uncertainty, limiting how effectively these tools could support decision-making if they were not accurately updated in real time.

#### Clinic Lead Artifacts Model

**Figure 3:**
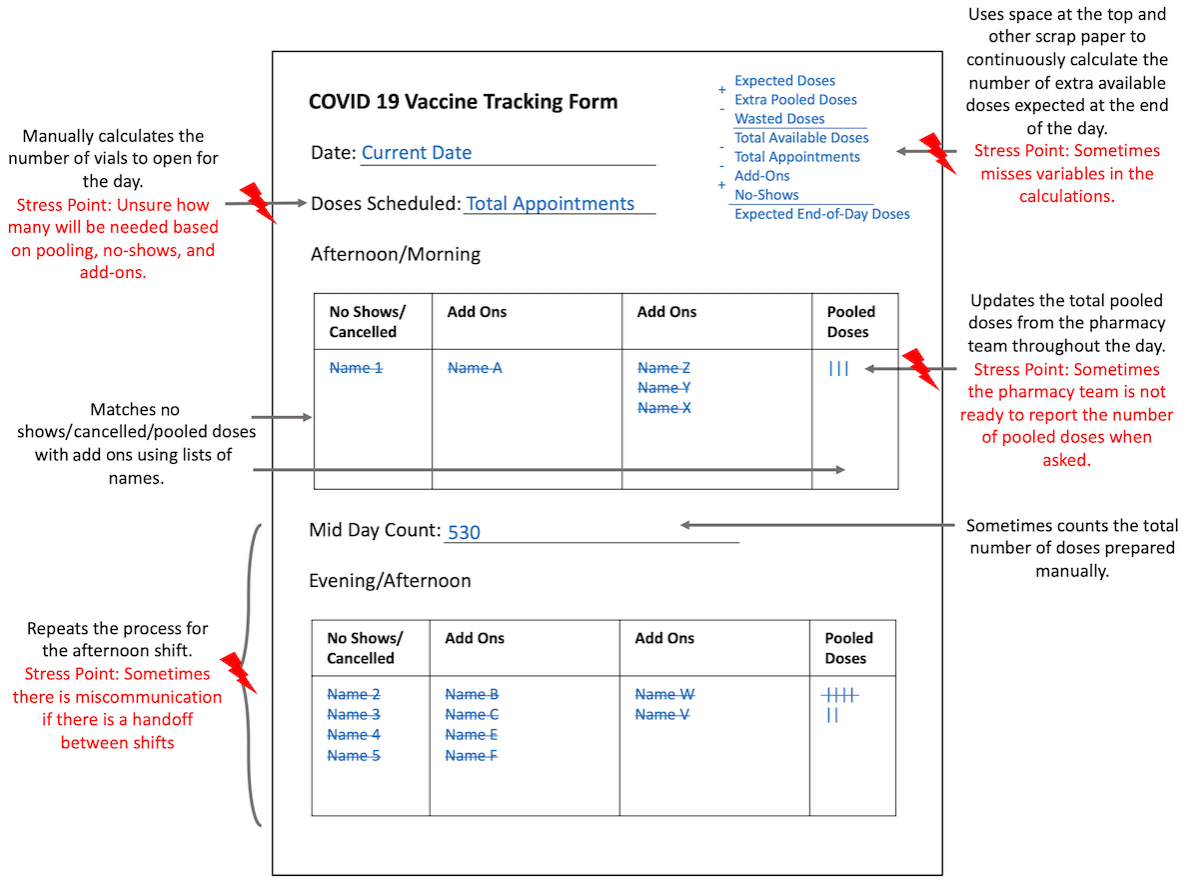
Clinic lead artifact model for vaccine and client tracking.

The clinic leads and superusers also developed artifacts ranging from paper-based tools to digital spreadsheets to track the number of clients expected to be immunized. In contrast to the vaccine lead artifact model, this analysis was consolidated through a paper-based representation. The paper-based model best depicts the mental models of clinic leads and superusers for client intake management (Figure 3).

The most critical challenge was using the client intake and vaccine preparation information to support decision-making on the number of vials to open and doses to prepare. This was partly due to uncertainties in client arrival behaviors. Timely updates from the vaccine lead on the status of pooling extra doses, which varied depending on the vaccine brand, lot number, and style of syringes and needles being used (ie, varying the amount of dead volume), also contributed to uncertainty. A simpler version of the client tracking paper artifact did not include information on total vials or doses, which was used by clinic leads, superusers, and other staff who did not hold responsibilities regarding vaccine preparation decisions.

While Figure 3 is a consolidated representation of the clinic lead artifacts used to track clients and doses, versions of this tool were also implemented in digital spreadsheets at clinics working with more than one vaccine brand. The primary benefits of the digital implementation were automating calculations and providing greater granularity of tracked information. For example, while some spreadsheets categorized no-shows, deferrals/refusals, immunization ineligibilities, and duplicate appointments into 1 category, others maintained these separate categorizations. Additional data included in digitalized versions were the number of client walk-ins and add-ons from staff or volunteers being immunized; clients that requested their information be excluded from the province’s immunization record website; the number of 5- and 7-dose vials; and wasted, pooled, and offsite doses brought to the clinic. A vaccine preparation rate calculation in response to the total number of immunizations over time was also included.

### Cultural Model

**Figure 4:**
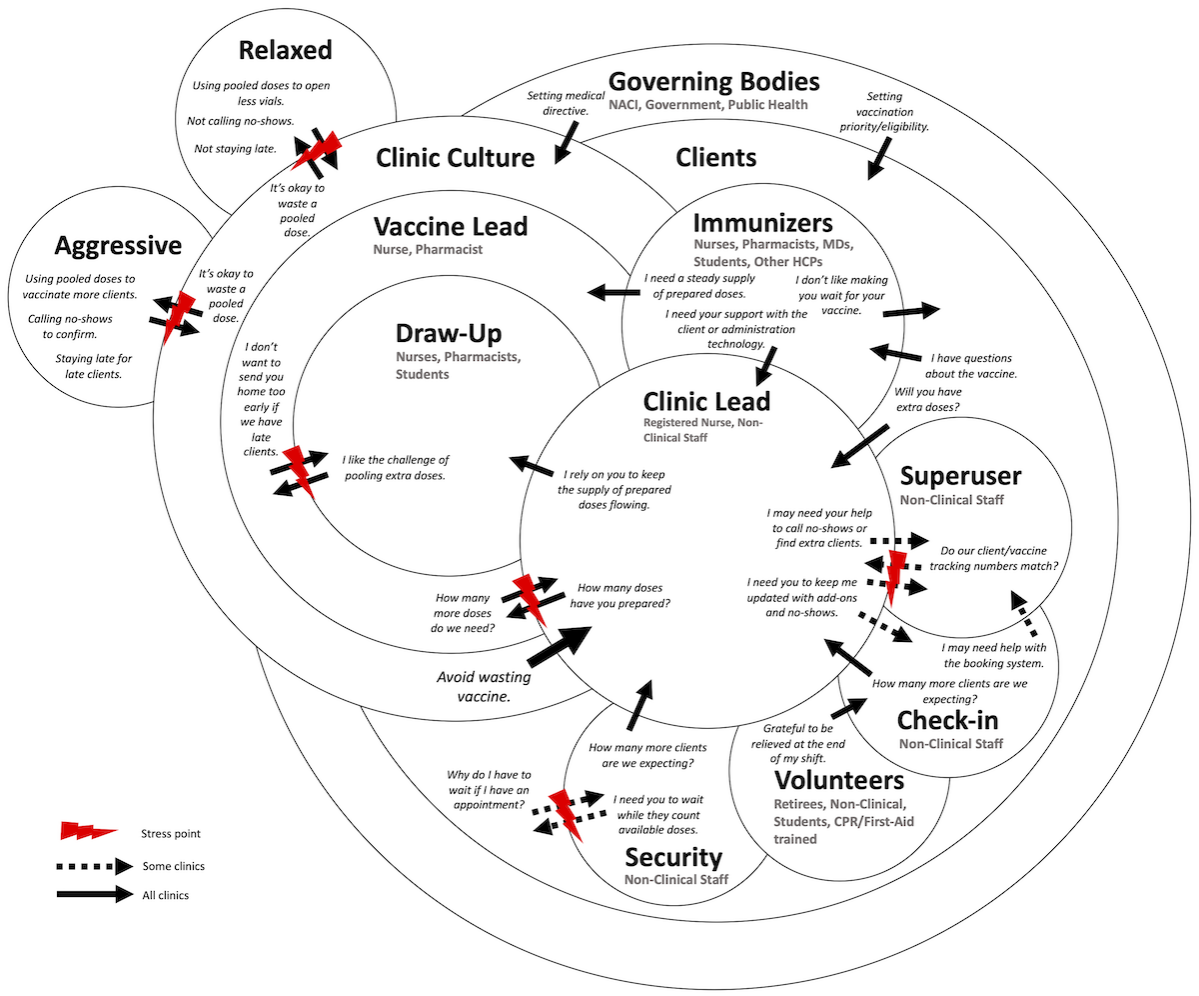
Consolidated cultural model for mass vaccination clinics. CPR: cardiopulmonary resuscitation; HCP: health care professional; MD: medical doctor; NACI: National Advisory Committee on Immunization.

Two workplace cultures were observed in the mass vaccination clinics: (1) the aggressive approach and (2) the relaxed approach (Figure 4). These cultures were primarily influenced by attitudes toward maximizing client throughput and managing vaccine waste, but also adapted in response to governing policies on vaccine preparation (eg, pooling residual doses), updated client eligibility, and vaccine scarcity. Client intake tracking remained the responsibility of the clinic lead in both approaches. However, the responsibility for tracking vaccine preparation and calculating the number of expected extra doses remaining at the end of the day fell on the clinic lead in the aggressive approach and the vaccine lead in the relaxed approach.

#### Aggressive Approach

The aggressive approach aimed to vaccinate as many clients as possible with the amount of available vaccine. The vaccine draw-up team would create pooled doses from residual volumes shortly after preparing the expected number of doses from each vial while generally opening most of the vials planned for the number of appointments. This approach was primarily observed at smaller clinics with enough vaccines for 1 day, and increased the stress and the satisfaction of vaccinating more eligible community members. In times of vaccine scarcity, it was sometimes considered wasting vaccine if there was no attempt to pool residual volumes. It was also seen as an exciting challenge by draw-up teams, which required developing skills and new techniques to retrieve the remaining volumes of the vaccine in the vials. Clinic leads also saw it as an exciting challenge to achieve a new daily vaccination record for the clinic. They called clients who did not attend their appointments to confirm whether they would arrive later for their vaccine. However, this approach could create stress about finding eligible clients for extra doses without keeping staff, immunizers, and volunteers beyond the clinic’s scheduled operating time.

#### Relaxed Approach

The relaxed approach aimed to vaccinate at maximum the number of scheduled clients, with the expectation of a decrease in this number throughout the day with no-shows or cancellations. This approach, used at larger clinics with more than a day’s worth of stored vials, additionally used residual pooling to open fewer vials. For example, 1 less vial was opened for the Pfizer-BioNTech vaccine for every 6 pooled doses. The vaccine was still treated like it was scarce, but there was less pressure to pool residual volumes.

As vaccine scarcity changed, clinics would decide to pool doses within their first batch of thawed vials or during the first half of the clinic, saving the remaining residual volumes for the end of the day. If clinic leads determined that they would fall short of doses, they would inform the vaccine leads to pool residual volumes from the vials that had not been pooled yet. The remaining residual volumes that were not pooled would be discarded. In larger clinics, it was also noted that clinic leads would call clients who did not show up for their appointment. However, this task was sometimes delegated to a superuser. This approach often resulted in fewer extra doses at the end of the clinic day. However, it remained a challenge for the draw-up team to know if they had enough residual vial volumes to meet client intake demand without having to start thawing more vials early enough to prevent delays, especially considering the expiration window.

### Physical Model

**Figure 5:**
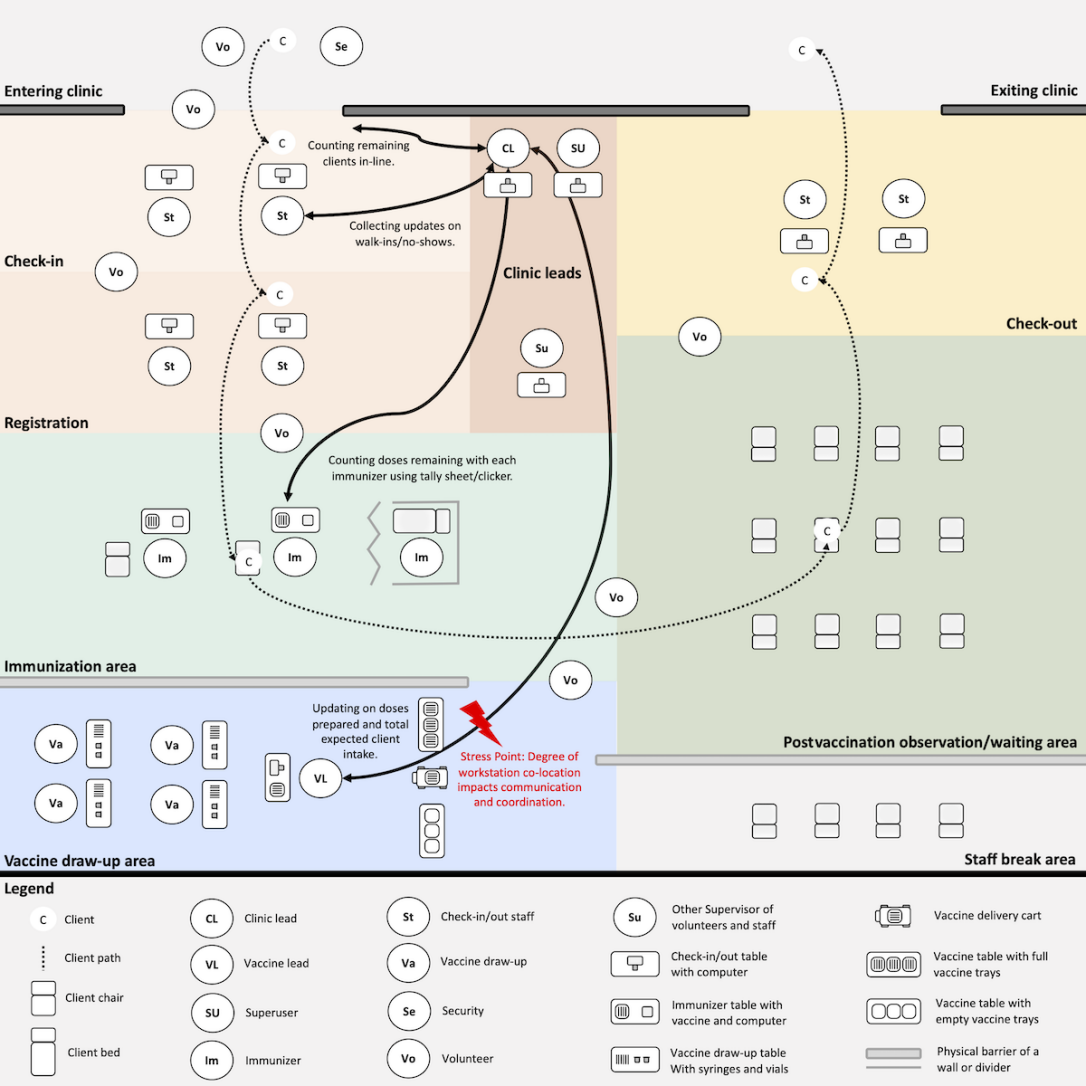
Consolidated physical model for mass vaccination clinics.

The mass vaccination clinics originally consisted of the following 6 stations for clients to move between: (1) COVID-19 symptom screening, (2) checking-in for the appointment, (3) registering their personal health information, (4) receiving their vaccine, (5) sitting in the postvaccination observation and waiting area, and (6) checking-out of the clinic, where they received their vaccination receipt and, if applicable, booked their next appointment (Figure 5). Depending on the physical environment, some clinics implemented each station within a large open space or within separate rooms.

Each clinic’s physical environment influenced where the clinic lead and the vaccine lead established their primary workstations. The larger the clinic, the more separated they were. Often, the vaccine lead worked near the immunization area, whereas the clinic lead maintained their workstation closer to the clinic entrance.

The team leads maintained frequent communication about vaccine preparation and client intake when their workstations were co-located. They were observed to make decisions quickly, and approaching each other for updates was less of an interruption of their work. It was easier to create shared awareness about the clinic’s status. However, when their workstations were not co-located, it was observed that frequent communication was more challenging. The greater physical distance appeared to reduce the efficiency of communicating updates and maintaining an accurate understanding of the clinic’s status. Strategies to overcome the physical space included writing on large whiteboards visible across the clinic and setting expectations for sharing updates at specific times.

### Decision Ladder Model

The primary decision-making activity identified within mass vaccination clinics surrounded the function of avoiding vaccine waste. It was critical to understand the state of the vaccine clinic concerning the number of expected end-of-day doses, which influenced the subsequent actions the team leads would take to reduce the number of wasted doses. The clinic leads determined how many extra clients would be required for extra doses, when to start looking for additional clients to ensure they would arrive before the end of the clinic day, and where to find eligible clients. The vaccine leads determined when to stop vaccine draw-up, if they should draw up doses from new vials, and if they should draw up doses from residual vial volumes to create pooled doses.

**Figure 6:**
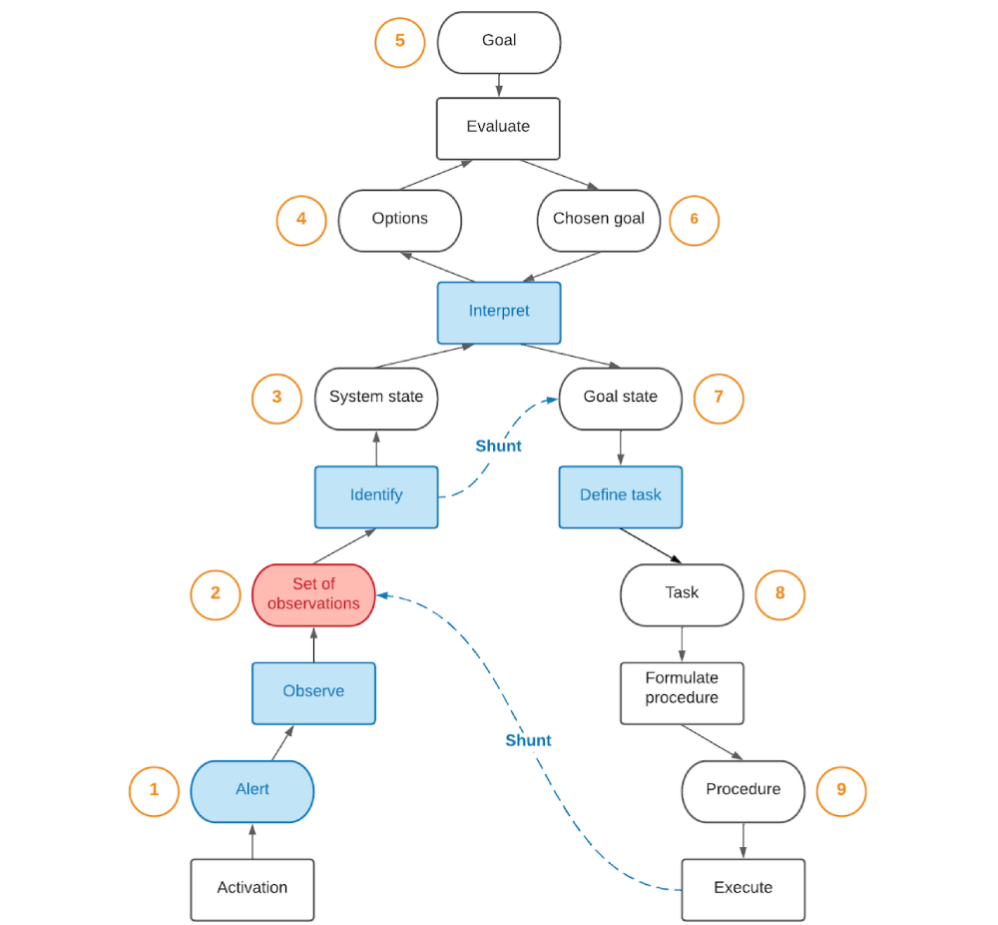
Consolidated decision ladder model for mass vaccination clinics.

In Figure 6, data processing activities and knowledge states in the decision ladder model that were influenced by uncertainty beyond a decision-maker’s control are highlighted in red. Additionally, specific knowledge states and data processes that would benefit from support through clinic process redesign or new technologies are highlighted in blue. Shunts were present between data processing activities and the system states that expert decision-makers effectively jumped between, indicating areas within the decision-making activity of avoiding vaccine waste where introducing supports may also assist novice decision-makers.

#### Step 1: Alerts

Alerts are provided by multiple sources as follows: (1) a clinic staff member, (2) a superuser, (3) a security staff member, (4) the vaccine lead, (5) the clinic lead, (6) an immunizer, (7) the booking website, and (8) the vaccine administration website. Alerts are often provided actively by the sources of the information once updates are available, or the decision-maker actively asks for new information. The alert state may be supported through technology by consolidating clinic information within a single location or digital database that each information source can access, along with establishing processes and expectations for when information sources shall alert the decision-maker of updates.

#### Step 2: Set of Observations

The objective of this step is to collect and understand information on total values from individuals who are collecting data concerning the following: appointments; add-ons or walk-ins; no-shows, cancellations, deferrals, refusals, or ineligibilities; opened vials; remaining vials; pooled or residual doses; doses in preparation; doses remaining with immunizers; expected residual doses; wasted doses; and administered doses.

Uncertainty exists with some of the collected data. The booking website must accurately represent the total number of appointments, no-shows, or cancellations, as undiscovered duplicate appointments will overestimate the number of required doses. Also, if there is a lack of coordination between individuals tracking walk-ins, there is a risk that these clients could be double counted.

Similar uncertainty exists with the accuracy of the vaccine administration website representing the total number of administered doses, which assumes immunizers entered the event correctly. Additionally, if an immunizer does not vaccinate a checked-in client due to ineligibility, they need to inform someone about the extra dose. Clients who do not want their information stored within the province’s online system will also not be included on the vaccine administration website and must be accounted for on a paper form.

Every event that impacts the number of required doses is essential information for the decision-maker. Therefore, this stage may also be supported by consolidating information within a single location, shared database, or collaborative interface.

Expert decision-makers may directly advance to this stage after executing tasks. After acting on a decision and updating the corresponding client intake or vaccine preparation information, they immediately understand the new state of the clinic concerning anticipated end-of-day extra doses.

#### Step 3: System State

At this stage, the decision-maker synthesizes the observed client intake and vaccine preparation information to determine how many available doses they expect to have at the end of the day. However, synthesizing data may be challenging if it dynamically changes. The decision-maker will often count the number of doses remaining with immunizers and the draw-up team to confirm the outcome of their calculations. Therefore, the system state may be supported by automaticity in the previous information processing stage, reducing the cognitive burden of manually performing calculations.

#### Step 4: Options

The decision-maker is generally faced with the following options: opening another vial, pooling residual vial volumes, finding clients for extra available doses, or sending additional clients away. The goal is to avoid significant perturbations in the state of the clinic (ie, needing significantly more doses or clients), requiring the reversal of the decision outcome if new information emerges.

#### Step 5: Goal

The overarching goal is to avoid vaccine waste. The potential for waste impacts overall stress with rising concerns about not having enough eligible clients to vaccinate. However, this objective is often balanced with the secondary aim of vaccinating as many eligible clients as possible, considering the potential impact on public health from contracting COVID-19 when unprotected by a vaccine.

#### Step 6: Chosen Goal

The decision-maker will decide how to balance maximizing vaccinations and minimizing wastage. However, they also aim to minimize extending clinic hours out of concern for burnout and client safety, and considering that extra clients are unlikely to approach the clinic for extra doses after the clinic is officially closed. The chosen approach depends on the culture of the clinic. 

#### Step 7: Goal State

The decision-maker will determine how many additional clients to call in to get vaccinated, how many extra vials to open (or not to open), and how many extra clients must be told that there are no additional extra doses. A shunt from the *Set of Observations* stage points toward the *Goal State* due to the potential for support through automation or expertise. Calculations to determine the total available extra doses can be automated or effectively determined by an expert decision-maker, indicating whether there is a deficit or surplus in vaccines and therefore a need to draw up more doses, call in more clients, or notify clients that there are no doses available for them. 

#### Step 8: Task

The decision-maker will decide when to notify additional clients about extra available doses, notify the draw-up team about opening extra vials, or stop opening vials, or choose when to tell clients that there are no more available doses. There is potential for supporting this stage by introducing technology that can provide real-time updates and calculations on the total available extra doses as new information emerges, given the dynamic nature of the clinic status. Real-time updates may improve decision-making efficiency.

#### Step 9: Procedure

Finally, the decision-maker will notify additional clients about extra available doses using public health and internal short-notice call lists, while also informing staff and volunteers within the vaccination clinic who are eligible. This process can become stressful if clients from these lists respond that they have already received their vaccine, do not answer their phone, or cannot arrive quickly. Consequently, the decision-maker may inform caregivers of clients within the vaccination clinic who are eligible for immunization. In dire situations, the decision-maker will look for extra clients outside the clinic by advertising extra doses to passers-by or within nearby commercial buildings. The final resort to avoid vaccine waste is to send extra doses to a larger clinic. Depending on the number of expected extra clients, the decision-maker may request the draw-up team to prepare more doses by thawing a new vial (if every dose can be administered) or pool extra doses. Other actions that can be taken to avoid vaccine waste are to stop opening vials and to open fewer vials closer to the end of the clinic.

## Discussion

### Principal Findings

The objective of this study was to systematically evaluate *work as done* in mass vaccination clinics using the systems approach of a modified contextual design framework, highlighting the relationships and challenges related to mass vaccination clinic flow, developed artifacts, culture, physical layouts, and decision-making. The application of the contextual design framework is discussed within the dynamic nature of the COVID-19 pandemic, consolidating information across different mass immunization environments while policies and procedures were changing. We also discuss recommendations for improving the design of mass vaccination clinics and the potential for integrating digital technologies to support workflow coordination and cognitive work.

### Consolidating Multiclinic Characteristics

Consolidated contextual design models are foundational to building technologies that can be better integrated into the workplace and can enhance the working environment [24]. The overall framework recognizes that introducing new technologies will inherently change the working environment; therefore, it is critical to holistically understand the working environment as it exists to provide recommendations and design solutions with a greater chance for user adoption [33]. While we modified the contextual design process to include control task analysis, the resulting decision ladder from this process enhances the understanding of cognitive processes, including differences between novice and expert decision-makers [36,39,40]. It also models how tasks and procedures are executed to achieve the desired system state [25,41].

In health care, an understanding of the contexts and processes of a work domain plays a significant role when implementing design recommendations or new technologies for health care professionals [42-44]. Successful implementation requires designing for the inherited context by matching the working culture, information needs, and work as done without introducing a significant workload [23,42,45]. It is also critical to recognize the importance of designing for humans as an adapting component in the system [23].

Only 1 descriptive report in the context of COVID-19 has provided a narrative description of vaccine clinic culture [12], pointing toward the unique challenges of designing for this health care context. In our study, the cultures of the observed mass vaccination clinics concerning role responsibility specificity, the approach for running the vaccination clinics, and the approach for handling end-of-day doses played critical roles in clinic workflow differences. Vaccine clinic cultures also indicated how workload stress might be distributed among decision-makers. Without a standardized approach to running mass vaccination clinics and individual differences between staff attitudes and beliefs across sites, this can pose a challenge for developing technological solutions that can be universally implemented to solve systemic inefficiencies.

Other nuances among mass vaccination clinics may additionally hinder the implementation of design recommendations and new technologies. This includes designing for inconsistent terminology and communicating the purposes behind recommendations when clinics approach mass immunization differently. For example, the language difference between clinics referring to extra doses as “pooled” or “residual” doses is an unsurprising inconsistency. Issues with terminology have been similarly identified in other health care contexts like medication prescribing [46]. The language inconsistencies observed in this study are likely due to the developing nature of the pandemic and different health care groups overseeing their respective clinics.

Performing contextual inquiry and design in real time has previously been successful in effectively developing health care technologies [47,48]. This study’s ethnographic approach captured the human factors necessary to understand system design within the disparate mass vaccination settings observed. The observed clinics in this study were united by the overarching goal of immunizing eligible clients and minimizing vaccine waste. Despite the cultural nuances, physical environments, and differences in staff responsibilities, the consolidated findings inform the development of generalized design recommendations, strategies, and new technologies to support these overarching objectives.

### Recommendations for Improving Mass Vaccination Clinics

The developed models from this study collectively identify the need to support the limitations of information certainty (Textbox 1) and information sharing among decision-makers (Textbox 2). While considering the potential variations in the approach to mass immunization, these recommendations are expected to be applicable to improve mass immunization clinics regardless of the nuances that make them unique.

**Mass vaccination clinic recommendation #1.**

Implement strategies and artifacts that reduce uncertainties for collecting and synthesizing client intake and vaccine preparation information required for end-of-day dose decision-making.

**Mass vaccination clinic recommendation #2.**

Improve data sharing and its collective interpretation among staff by co-locating workstations and implementing collaborative artifacts that support shared situational awareness about the state of the clinic.

First, information uncertainty creates a high gain system (ie, where sudden changes can result in surplus vaccines) and impacts decision-making processes, especially in health care [49]. This uncertainty results in behaviors that aim to avoid decision regret and may be suboptimal for overall system performance [50]. Therefore, mass vaccination clinics should be designed to support certainty in information about client intake and vaccine preparation to reduce this associated stress. One potential strategy includes always using an appointment-based system to increase information certainty for how many clients are expected throughout the day and the number of doses to prepare. As previously identified, appointment-based systems double as a strategy to prevent bottlenecks in clinic flow by controlling the maximum client intake volume [51]. A layer of certainty to client intake can also include assigning staff to call no-shows and confirm their arrival, as identified by the aggressive cultural approach to operating a clinic. From the vaccine lead perspective, strategies to reduce uncertainty include the early identification of the expected number of doses per multidose vial, using the style of syringes and needles provided for the draw-up, while also accounting for potential equipment shortages that may influence the total amount of expected doses (ie, changes in syringe dead volume). From the relaxed approach, reducing the number of new vials required for every vial’s worth of pooled residual doses may decrease the risk of a surplus or scarcity of doses in a future state of the clinic.

Digital technologies can also reduce information uncertainty in mass vaccination clinics to support overall system resilience while potentially being broadly adopted by frontline workers, as seen in prior mass immunization contexts [52]. Information certainty can be improved within data processing tasks by reducing human error, which can support the accuracy of the calculations required to determine the numbers of anticipated end-of-day extra doses, remaining clients, and remaining doses through automation in real time. Automating the calculations needed to operate a mass vaccination clinic may standardize end-of-day dose decision-making regardless of the clinic culture. Subsequently, if digital tools can be widely adopted, this may standardize data processing activities across more than one clinic.

Second, information sharing among data source stakeholders is essential for successfully operating a mass vaccination clinic. Shared information supports decision-making tasks through a collective understanding of the dynamic state of the clinic and how it might change in the future (ie, the degree of vaccine excess or vaccine shortage). Shared interprofessional decision-making is fundamental in health care [49]. In the context of mass vaccination clinics, this process occurs between the clinic lead and the vaccine lead. It is critically essential to have the information and the skills that enable shared knowledge of the current and future states of the clinic when changes in client intake and vaccine preparation occur.

As observed in this study, increased co-location is one way to support shared awareness between staff managing client intake and vaccine preparation information, and decision-making. Additionally, establishing communication expectations for when and how to update information between staff members was observed to support situational awareness when followed (eg, updating on the hour using a standard template). While establishing clinics in large open spaces has been previously shown to improve workflow efficiencies with clients due to greater flexibility in workstation placement [20], clinics operating out of such environments should consider primary workstation placements that reduce the physical separation of decision-makers and the data they need.

However, the rigidity of the physical environment may prevent co-locating workstations, and decision-makers may be moving throughout the clinic, creating a cognitive burden to gather and synthesize information partly in isolation. One solution is to develop and implement collaborative artifacts that support situational awareness through real-time shared databases, computerized applications, or large displays (eg, a large whiteboard or poster that can be viewed across the clinic). This would reduce the need to *physically* gather dynamic data, similar to the workflows within intensive care units [53]. In vaccine clinics, implementing artifacts that automatically synthesize information in a standard way through a shared interface would further bridge the artifact silos that currently exist. If intuitively displayed, this would support a shared understanding of the state of the clinic when co-locating workstations is infeasible.

### Strengths and Limitations

This is the first study to apply a human systems approach to mass vaccination clinics during the COVID-19 pandemic with an interdisciplinary research team. However, while the comprehensive data collected from this study included the Region of Waterloo in Ontario, Canada, the results have not been validated in other regions or within resource-limited settings. Although the researchers confirmed the raw observation data with staff, only the research team reviewed the resulting models.

While this study captures important high-level insights during a critical period of the COVID-19 pandemic in the Region of Waterloo, we recognize that it does not entirely capture the complexities of all mass vaccination environments or precisely replicate real-world scenarios throughout the pandemic. Therefore, further research should examine the inclusion of additional complexities in the developed models and their application beyond mass vaccination clinics in the Region of Waterloo. Future work can also evaluate the recommendations on workflow coordination, stress, and decision-making. Finally, microergonomic analyses aimed at improving specific processes in mass vaccination clinics could support the optimal design of public health documentation and workstations for vaccine draw-up.

### Conclusions

This study provides a holistic representation of the working environments in mass vaccination clinics within the Region of Waterloo, highlighting work as done and human factors challenges in the following human systems models: flow, artifacts, cultural, physical, and decision-making. A systems analysis using human factors methodologies has provided a critically important lens on mass vaccination clinics in the context of COVID-19. The collaboration between human factors researchers and health care professionals in this study has also shown essential advantages for effectively understanding and modeling this complex health care environment and providing important recommendations to improve this complex tightly coupled system. While this research provides a fundamental understanding of mass vaccination clinics centered around frontline workers, further collaborative research that bridges human factors and health care professionals can advance the scope of knowledge on improving this evolving global public health activity.

## Appendix

Multimedia Appendix 1 Observed workflow-related tasks and responsibilities in mass vaccination clinics.

Multimedia Appendix 2 A detailed version of the consolidated flow model for mass vaccination clinics.
