# Supplementary material for: Understanding Human Factors Challenges on the Front Lines of Mass COVID-19 Vaccination Clinics: Human Systems Modeling Study
Source: JMIR Hum Factors. 2022 Nov 10;9(4):e39670. doi: 10.2196/39670 (PMC9693702; doi:10.2196/39670)
Supplement: Multimedia Appendix 1 [file humanfactors_v9i4e39670_app1.pdf]

Staff Role	Observed Workflow Related Tasks & Responsibilities
**Clinic Lead**	• Tracking the total number of appointments.<br>• Tracking the total number of vaccinations given.<br>• Determining if a client will likely not arrive for their appointment (i.e., no-shows).<br>• Tracking appointment cancellations.<br>• Tracking add-ons or walk-ins.<br>• Tracking vaccine brand refusals or ineligibilities.<br>• Tracking doses received from other clinics.<br>• Updating the Vaccine Lead on the total number of clients.<br>• Calculating the number of expected doses to prepare.<br>• Counting and tracking the number of vials stored.<br>• Deciding if an extra client can be an add-on.<br>• Handling medical emergencies/other situations.<br>• Taking on the responsibilities of a 'Superuser' if this role is not filled by someone else at the clinic.
**Vaccine Lead**	• Counting the number of available vials stored.<br>• Assigning team members to prepare the vaccine.<br>• Removing vials from the fridge and monitoring expiry.<br>• Checking prepared syringes to confirm volume and identify bubbles.<br>• Tracking the total number of prepared doses.<br>• Updating the Clinic Lead or 'Superuser' on the number of doses prepared.<br>• Tracking the total number of extra doses (e.g., pooling extra doses from residual volumes and unused doses).<br>• Tracking the total number of wasted doses (e.g., dropped vial/dose).<br>• Tracking the doses received from other clinics.<br>• Deciding when to prepare more doses to meet client influx to the clinic.<br>• Calculating the number of expected doses to prepare.
**Vaccine Draw-up**	• Preparing single dose syringes for Immunizers.<br>• Immunizing clients if additional Immunizers are needed.<br>• Updating the Vaccine Lead about the number of doses they have prepared for each vial opened.
**Immunizer**	• Requesting more doses from the vaccine preparation team to their station.

	• Entering the vaccine administration events into the online vaccination record.<br>• Informing the Clinic Lead about vaccine brand refusals or ineligibilities.<br>• Informing the Vaccine Lead about issues with syringes or wasted doses.
**Superuser**<br>(Clinics #2, #3, #6)	• Tracking the total number of appointments.<br>• Determining if a client will likely not arrive for their appointment (i.e., no-shows, cancellations).<br>• Tracking add-ons or walk-ins.<br>• Calculating the number of expected doses to prepare.<br>• Fixing issues with the appointment booking and vaccine administration websites.
**Check-in Staff**	• Confirming client appointment arrivals.<br>• Determining if a client will likely not arrive for their appointment (i.e., no-shows, cancellations).<br>• Tracking add-ons or walk-ins.<br>• Reporting their tracked client intake numbers to a 'Superuser' or Clinic Lead.
**Volunteer**	• Monitoring for issues with clinic flow and alleviating client backlogs by finding solutions to control flow.<br>• Ensuring clients maintain distancing and masking.<br>• Screening clients for COVID-19 symptoms.<br>• Sanitizing surfaces after use.<br>• Responding to client questions about clinic flow, available vaccine types, and waiting times.<br>• Relaying client questions about extra doses and vaccine brand preferences to Security Staff, Clinic Staff or Clinic Leads.<br>• Asking how many clients remain to arrive for their vaccine at the end of the day.<br>• Supporting anxious clients to remain calm.
**Client**	• Arriving for their appointments early, on time or late.<br>• Arriving without an appointment.<br>• Asking about extra dose availability.<br>• Cancelling their appointment.<br>• Not showing up for their appointment.<br>• Requesting a specific vaccine brand.<br>• Being ineligible for vaccination.
