# Supplementary figures and images for: Understanding Human Factors Challenges on the Front Lines of Mass COVID-19 Vaccination Clinics: Human Systems Modeling Study
Source: JMIR Hum Factors. 2022 Nov 10;9(4):e39670. doi: 10.2196/39670 (PMC9693702; doi:10.2196/39670)

# Vaccine Preparation

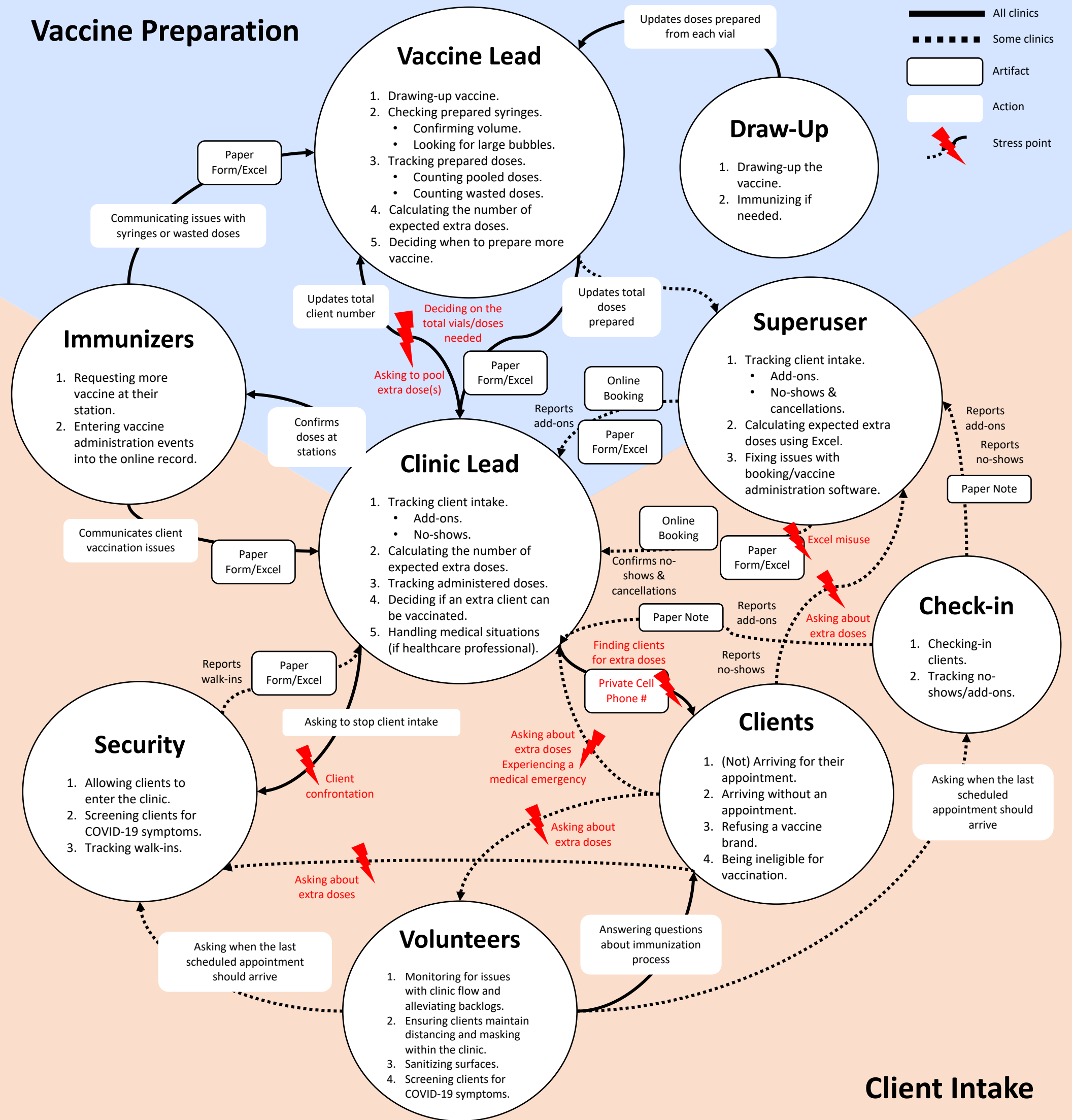

Supplement: Multimedia Appendix 2 [file humanfactors_v9i4e39670_app2.pdf]
